# The role of cyanoalanine synthase and alternative oxidase in promoting salt stress tolerance in *Arabidopsis thaliana*

**DOI:** 10.1186/s12870-023-04167-1

**Published:** 2023-03-27

**Authors:** Fei Xu, Ye Peng, Zheng-Quan He, Lu-Lu Yu

**Affiliations:** 1 College of Biological and Pharmaceutical, China Three Gorges University, Yichang, 443002 China; 2 School of Life Science and Biotechnology, Wuhan University of Bioengineering, Wuhan, 430415 China

**Keywords:** Salt Stress, Cyanide, Hydrogen sulfide, β-cyanoalanine synthase, Cellular respiration

## Abstract

**Background:**

Cyanide is a toxic chemical that inhibits cellular respiration. In plants, cyanide can be produced by themselves, especially under stressful conditions. Cyanoalanine synthase (CAS) is a key enzyme involved in plant cyanide detoxification. There are three genes encoding CAS in *Arabidopsis thaliana*, but the roles of these genes in the plant’s response to stress are less studied. In addition, it is known that alternative oxidase (AOX) mediates cyanide-resistant respiration, but the relationship between CAS and AOX in regulating the plant stress response remains largely unknown.

**Results:**

Here, the effects of the overexpression or mutation of these three *CAS* genes on salt stress tolerance were investigated. The results showed that under normal conditions, the overexpression or mutation of the *CAS* genes had no significant effect on the seed germination and growth of *Arabidopsis thaliana* compared with wild type (WT). However, under 50, 100, and 200 mM NaCl conditions, the seeds overexpressing *CAS* genes showed stronger salt stress resistance, i.e., higher germination speed than WT seeds, especially those that overexpressed the *CYS-C1* and *CYS-D1* genes. In contrast, the seeds with *CAS* gene mutations exhibited salt sensitivity, and their germination ability and growth were significantly damaged by 100 and 200 mM NaCl. Importantly, this difference in salt stress resistance became more pronounced in *CAS*-OE, WT, and mutant seeds with increasing salt concentration. The *CAS*-OE seeds maintained higher respiration rates than the WT and CAS mutant seeds under salt stress conditions. The cyanide contents in *CAS* mutant seeds were approximately 3 times higher than those in WT seeds and more than 5 times higher than those in *CAS*-OE seeds. In comparison, plants overexpressing *CYS-C1* had the fastest detoxification of cyanide and the best salt tolerance, followed by those overexpressing *CYS-D1* and *CYS-D2*. Furthermore, less hydrogen sulfide (H_2_S) was observed in *CAS*-OE seedlings than in WT seedlings under long-term salt stress conditions. Nonetheless, the lack of AOX impaired *CAS*-OE-mediated plant salt stress resistance, suggesting that CAS and AOX interact to improve salt tolerance is essential. The results also showed that CAS and AOX contributed to the reduction in oxidative damage by helping maintain relatively high levels of antioxidant enzyme activity.

**Conclusion:**

In summary, the findings of the present study suggest that overexpression of Arabidopsis *CAS* family genes plays a positive role in salt stress tolerance and highlights the contribution of AOX to CAS-mediated plant salt resistance, mainly by reducing cyanide and H_2_S toxicity.

**Supplementary Information:**

The online version contains supplementary material available at 10.1186/s12870-023-04167-1.

## Background

Soil salinization is a growing problem for agriculture worldwide. It harms approximately 900 million ha of land, which is close to 20% of the worldwide land area and nearly half of the total arable land irrigated globally [1]. Increased soil salt concentrations decrease the ability of plants to take up water and inhibit seed germination and plant growth, development, and yield [2]. Therefore, increasing plant salt tolerance is very important to achieve sustainable agriculture and ensure that global food demand is met. Notably, stressful conditions such as salt stress can trigger cellular damage including increased reactive oxygen species (ROS) and the accumulation of toxic substances such as cyanide.

Cyanide is produced by higher plants via multiple metabolic pathways. The two most prevalent sources of endogenous cyanide are the turnover of cyanogenic glycosides or cyanolipids [3, 4] and ethylene biosynthesis [5]. Plants produce cyanide when synthesizing the hormone ethylene from the precursor 1-aminocyclopropane-1-carboxylic acid (ACC). However, cyanide is well known as an inhibitor of cytochrome *c* oxidase of the mitochondrial electron transport chain; furthermore, it also inhibits other enzymes, notably catalase, peroxidase, nitrate/nitrite reductase, superoxide dismutase, and Rubisco [3, 6]. To prevent self-poisoning, plants maintain an endogenous cyanide detoxification pathway, the cyanoalanine synthase (CAS; EC 4.4.1.9) pathway [7, 8]. Under the catalysis of CAS, cyanide reacts with cysteine to form hydrogen sulfide (H_2_S) and β-cyanoalanine and is then converted to either asparagine or aspartate in conjunction with ammonia by a dual-function nitrile hydratase/nitrilase (EC 3.5.5.1) [9, 10].

Recent evidence suggests that the CAS pathway plays a crucial role in plant response and in acclimation to abiotic stress. For instance, a study by Machingura et al. [7] demonstrated that the CAS pathway is responsive to water deficit, and the capacity to remove cyanide generated during water deficit may contribute to tolerance to this stress in Arabidopsis. Our previous studies have also shown that enhancing the expression of the CAS enzyme can significantly improve plant tolerance to salt stress in tobacco [11, 12]. However, the improvement in plant stress resistance by CAS relies on assistance from the alternative oxidase (AOX) pathway, indicating that the endogenous plant cyanide detoxification system appears to have limited capacity and evolved principally to deal with the relatively small amounts of cyanide produced during metabolism [6, 11]. AOX is an enzyme that forms part of the electron transport chain in mitochondria, which mediates cyanide-resistant respiration and is critical under conditions that impair the cytochrome pathway [13]. In addition, plants are capable of assimilating exogenous cyanide, and experiments with wheat have shown that this assimilation rate increases under nitrogen limiting conditions [14, 15]. Nevertheless, the effectiveness of cyanide assimilation in the response of plants to stress, such as salt stress, seems to be limited. In short, the existence of the CAS pathway in the cyanide detoxification process is necessary and important, and the mechanism of its participation in the response to adverse stress needs further study.

In Arabidopsis, there are three genes encoding CAS, namely, *CYS-C1* (At3g61440), *CYS-D1* (At3g04940), and *CYS-D2* (At5g28020) [16–18]. The most abundant CAS enzyme is CYS-C1, which is localized in the mitochondria and contributes most of the CAS activity in root and leaf tissue [16, 19]. The isoforms CYS-D1 and CYS-D2 are localized in the cytosol and are much less abundant than CYS-C1 [16]. It was shown that T-DNA insertion mutants of mitochondrial CYS-C1 conferred a strong inhibition of root hair development [16], increased susceptibility to the necrotrophic fungus *Botrytis cinerea*, and increased tolerance to the biotrophic *Pseudomonas syringae* pv. tomato DC3000 bacterium and *Beet curly top virus* in *Arabidopsis thaliana* [20]. However, the role of the *CAS* gene family in cyanide detoxification remains to be clarified, especially in plants with mutations in one of these genes, which have been confirmed to grow normally under nonstress conditions. This suggests that other enzymes might be involved in or assist in cyanide detoxification.

The same amount of H_2_S is produced when cyanide reacts with cysteine under the catalysis of CAS. In this regard, whether there will be excess H_2_S production in CAS-mediated plant stress resistance remains to be clarified. H_2_S is also an inhibitor of mitochondrial respiration, and its inhibitory site is the same as that of cyanide, acting on complex IV in the respiratory chain. Therefore, the metabolic balance between cyanide and H_2_S needs to be further studied under stress conditions. This study aimed to further our understanding of the specific function of the *CAS* gene family in the response of plants to salt stress. On this purpose, *CYS-C1*-, *CYS-D1*-*,* and *CYS-D2*- overexpressing transgenic lines and their T-DNA insertion mutants were used to compare salt stress resistances. The dynamic changes in cyanide and H_2_S were also investigated.

## Results

### Overexpression of *CAS* genes improves seed germination and growth under salt stress conditions

**Fig. 1:**
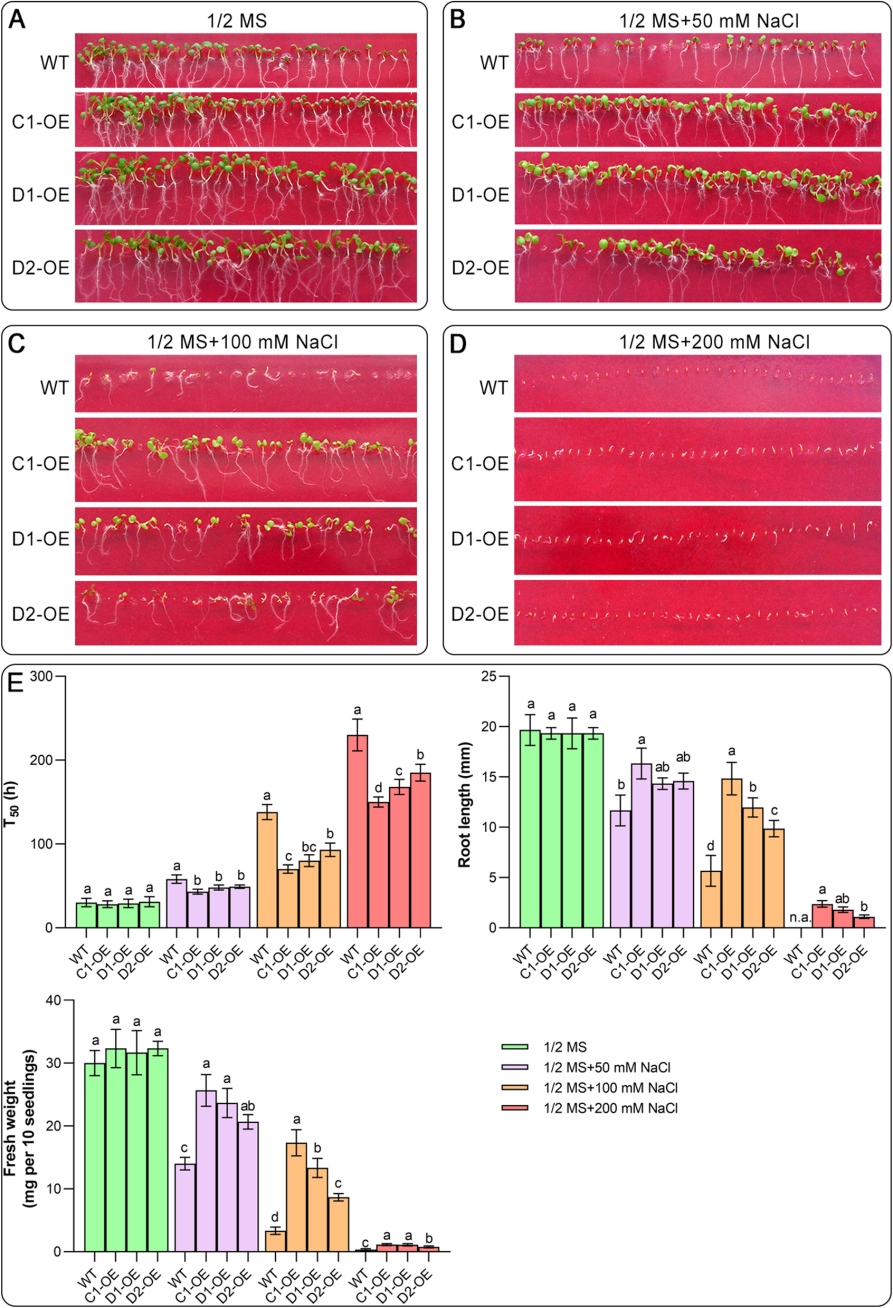
Effects of *CAS* gene overexpression on seed germination and growth. WT and *CAS*-OE seeds were sown on 1/2 MS medium without NaCl (**A**) or with 50 mM NaCl (**B**), 100 mM NaCl (**C**), or 200 mM NaCl (**D**), and seed germination and growth are shown after seven days of incubation. **E** The germination speed (T_50_), root length, and fresh weight were compared between different samples under normal and salt stress conditions. Data are the means ± SD of five independent experiments. Different lowercase letters above the bars represent significant differences according to post hoc analysis (Tukey’s HSD, *P* < 0.05). The following abbreviations for *CAS* gene overexpression were used for labeling in this figure and the following figures: C1-OE, CYS-C1-OE; D1, CYS-D1-OE; D2, CYS-D2-OE

To investigate the effects of *CAS* gene overexpression and mutation on Arabidopsis salt tolerance, the seeds were sown on media containing 1/2 MS (control) and different concentrations of NaCl (50, 100, and 200 mM NaCl). The results showed that the germination of the seeds overexpressing *CAS* genes was not significantly different from that of the wild type (WT) under normal conditions (Fig. 1A). However, under salt stress, seeds overexpressing *CAS* genes showed a faster germination rate and stronger salt adaptation than WT seeds (Fig. 1B-D). The root length and fresh weight of plants overexpressing *CAS* genes were also significantly higher than those of WT plants under 50 and 100 mM NaCl conditions, especially those overexpressing the *CYS-C1* gene (Fig. 1E). Notably, with increasing NaCl concentration, the salt tolerance of seeds overexpressing the *CAS* genes was also attenuated, especially with the 200 mM NaCl treatment (Fig. 1D).

Similar to *CAS*-OE seeds, the germination rate of seeds with T-DNA insertion mutants of the *CAS* gene family was not significantly different from that of WT seeds under normal conditions (Fig. S2). When the seeds were treated with different concentrations of NaCl, however, the seed germination ability and plant growth of *CAS* mutants were significantly impaired, most notably in *cys*-*c1* mutant seeds (Fig. S2). These results further suggest the importance of CYS-C1, a member of the Arabidopsis CAS enzyme, in the process of resistance to salt stress.

### Overexpression of *CAS* genes enhances seedling salt stress tolerance

**Fig. 2:**
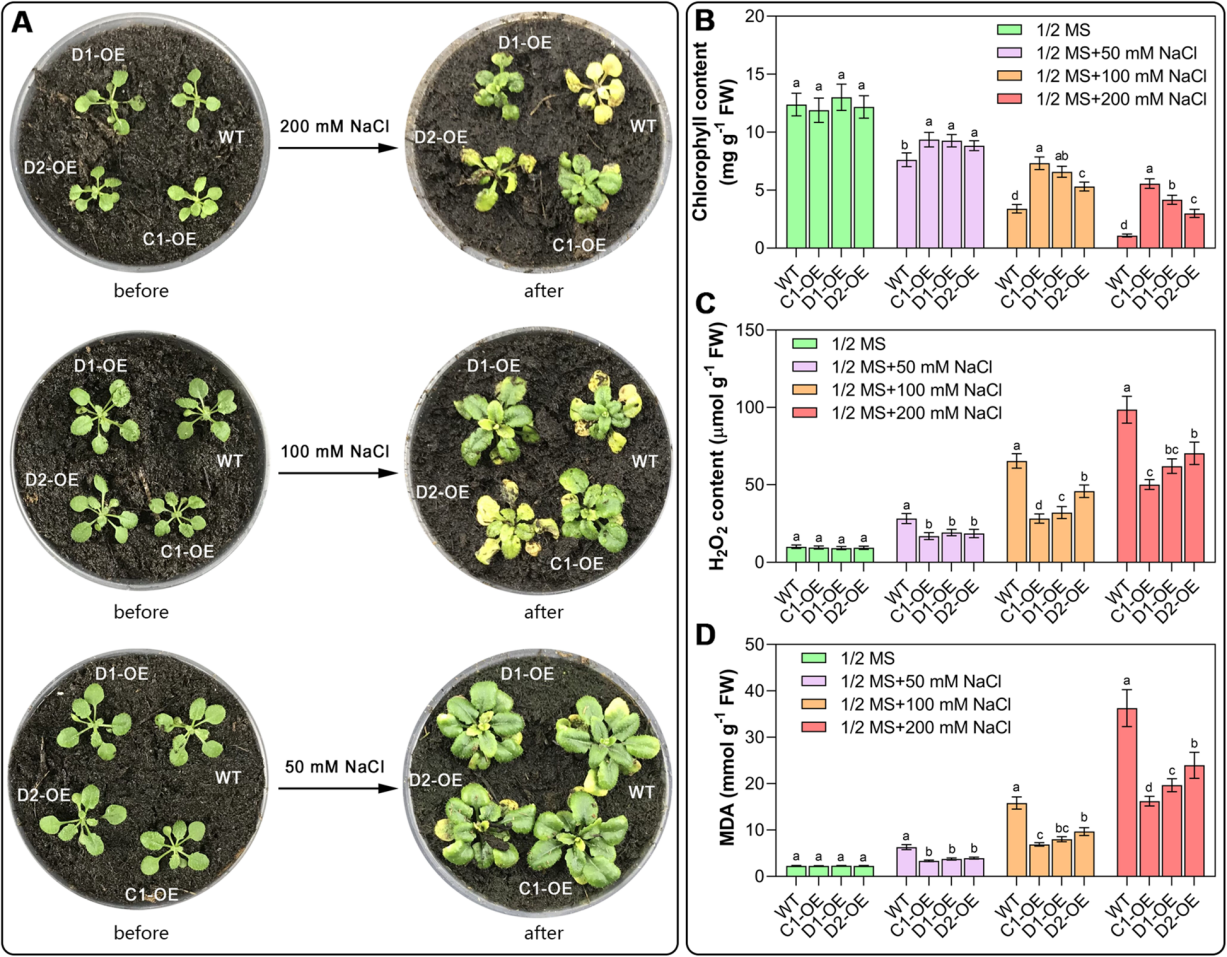
Effects of *CAS* gene overexpression on salt tolerance of Arabidopsis seedlings. For this experiment, 3-week-old WT and *CAS*-OE seedlings were irrigated with different concentrations of NaCl. After 14 days of salt stress treatment, seedling growth (**A**), chlorophyll content (**B**), H_2_O_2_ content (**C**), and MDA content (**D**) were compared between WT and *CAS*-OE plants. Data are the mean ± SD of five independent experiments. Different lowercase letters above the bars represent significant differences according to post hoc analysis (Tukey’s HSD, *P* < 0.05)

We next comparatively analyzed the effects of *CAS* gene overexpression and mutation on Arabidopsis seedling growth under normal and salt stress conditions. As shown in Fig. 2, when the WT seedlings were exposed to the 50, 100, and 200 mM NaCl treatments, the growth of the plants gradually deteriorated, with smaller plant sizes and smaller and yellow leaves, which became more obvious at higher salt concentrations. In comparison, the plants overexpressing the *CAS* genes showed better salt stress tolerance than the WT plants, regardless of whether lower (50 mM NaCl) or higher (200 mM NaCl) salt concentrations were applied. It should be noted that *CYS*-*C1*-OE seedlings showed the most prominent salt stress tolerance, followed by *CYS*-*D1*-OE and *CYS*-*D2*-OE seedlings under 100 mM and 200 mM NaCl conditions (Fig. 2B-D). Likewise, *CAS* mutants, including *cys-c1*, *cys-d1,* and *cys-d2*, showed significant growth inhibition, especially under the 100 mM and 200 mM NaCl treatments (Fig. S3).

Measurement of H_2_O_2_, MDA, and chlorophyll contents showed that salt stress increased the accumulation of H_2_O_2_ and MDA and accelerated the degradation of chlorophyll in WT seedlings, and these phenomena were more obvious under higher salt stress conditions (100 mM and 200 mM NaCl) (Fig. 2B-D). In comparison, the salt stress-induced damage to the *CAS*-OE seedlings was milder under 50 mM NaCl conditions compared to that of the WT seedlings (Fig. 2C, D). With increasing salt concentrations, the growth of *CAS*-OE seedlings was further inhibited, accompanied by chlorophyll degradation, i.e., yellowing of leaf margins (Fig. 2B). Consistent with the observed weaker plant growth, more stress injuries, including chlorophyll degradation and increased MDA content, were detected in plants with *CAS* gene mutations under all salt treatment conditions (Fig. S3).

### Overexpression of *CAS* genes reduces cyanide accumulation and helps maintain antioxidant activity

**Fig. 3:**
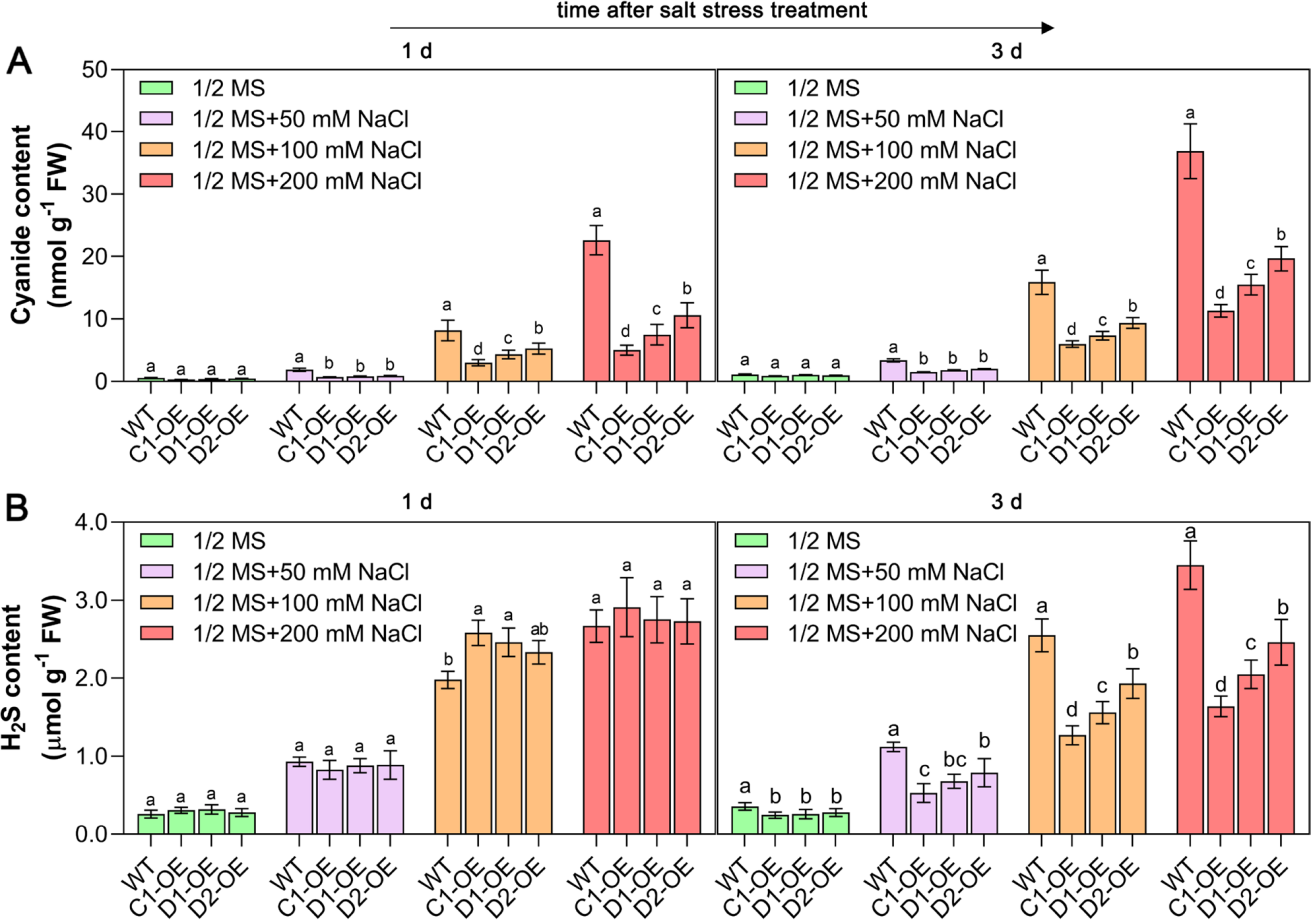
Comparison of cyanide and H_2_S contents between WT and *CAS*-OE seedlings. For this experiment, 3-week-old seedlings were treated with different concentrations of NaCl, and the cyanide (**A**) and H_2_S (**B**) contents were then measured during the first few days (days 1 and 3) of salt stress treatment. Data are the mean ± SD of five independent experiments. Different lowercase letters above the bars represent significant differences according to post hoc analysis (Tukey’s HSD, *P* < 0.05)

Since *CAS* genes are responsible for cyanide metabolism, the changes in cyanide contents between WT and *CAS*-OE seedlings under normal and salt stress conditions were investigated. *CAS* gene overexpression resulted in less cyanide accumulation in Arabidopsis seedlings under salt-stressed conditions than in WT seedlings, although the difference in cyanide content between them was less pronounced under normal conditions (Fig. 3A). Moreover, it should be noted that the cyanide content in WT Arabidopsis seedlings increased gradually with increasing salt concentration, as the amount of cyanide in the seedlings treated with 200 mM NaCl was nearly 10 times higher than that in the seedlings under normal conditions (Fig. 3A). In contrast, salt stress did not induce massive cyanide accumulation in *CYS*-*C1*-OE seedlings (Fig. 3A), although 200 mM NaCl treatment induced damage to the seedlings (Fig. 2). In addition, there was higher cyanide accumulation in *CYS*-*D1*-OE and *CYS*-*D2*-OE seedlings than in *CYS*-*C1*-OE seedlings (Fig. 3A). The difference in cyanide content between *CYS*-*C1*-OE and *CYS*-*D2*-OE was nearly threefold, which may be one of the reasons why *CYS*-*C1*-OE seedlings showed the most salt-tolerance.

Additionally, significant increases in the activities of antioxidant enzymes, including SOD, CAT, APX, and GPX, were observed in *CYS*-*C1*-OE, *CYS*-*D1*-OE, and *CYS*-*D2*-OE seedlings but not in WT seedlings after salt stress treatment for 1 day (Fig. S4). Under salt stress on the 3^rd^ day, the differences in the activities of antioxidant enzymes between *CAS*-OE and WT seedlings were more pronounced, and the highest activities were observed in *CYS*-*C1*-OE seedlings (Fig. S4). It should be noted that the activity of antioxidant enzymes in the seedlings overexpressing *CAS* genes remained at a high level under the 50 mM and 100 mM NaCl treatments but decreased significantly under the 200 mM NaCl treatment with increasing stress time (Fig. S4).

Considering that H_2_S is produced when cyanide is detoxified by the CAS enzyme, we next determined the change in H_2_S content in plants under normal and stress conditions. It is interesting to note that there were no significant differences in H_2_S content between WT and *CAS*-OE seedlings under normal and 50 mM NaCl conditions (Fig. 3B). However, when 100 mM NaCl was applied to the seedlings, higher H_2_S content was detected in *CAS*-OE seedlings than in WT seedlings on the first day, whereas lower H_2_S content was observed after 3 days of salt treatment. Likewise, the accumulated H_2_S content in *CAS*-OE seedlings was significantly lower than that in WT seedlings when they were treated with 200 mM NaCl for 3 days (Fig. 3B). In comparison, among *CAS*-OE plants, *CYS*-*C1*-OE plants accumulated the least amount of H_2_S, followed by *CYS*-*D1*-OE and *CYS*-*D2*-OE plants (Fig. 3B).

### Overexpression of *CAS* genes alleviates respiratory repression with the assistance of AOX

**Fig. 4:**
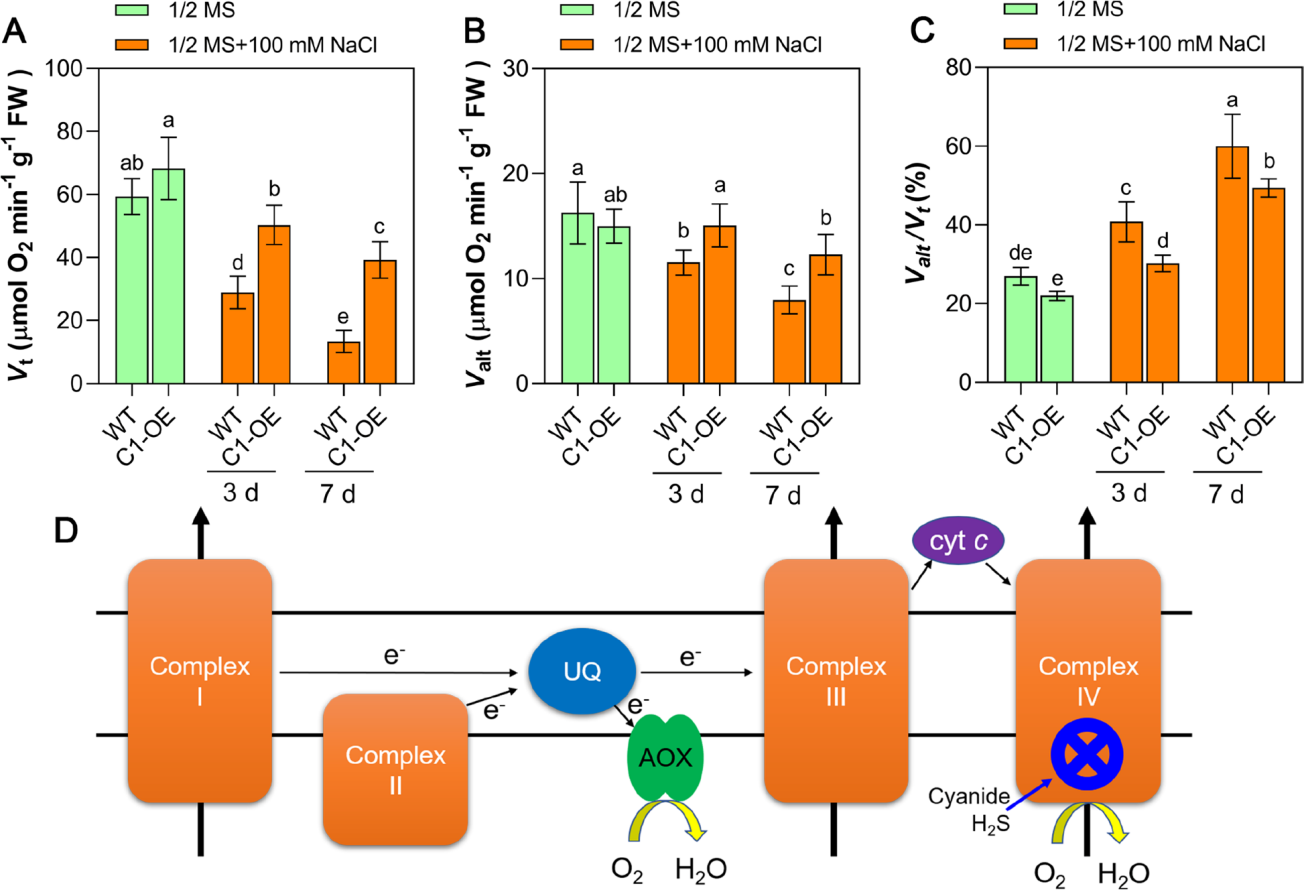
Comparison of respiration rates between WT and C1-OE seedlings. For this experiment, 3-week-old seedlings were treated with 100 mM NaCl, and then the *V*_t_ (**A**), *V*_alt_ (**B**), and *V*_alt_/*V*_t_ (**C**) ratio were compared between WT and C1-OE seedlings. *V*_t_, total respiration; *V*_alt_, AOX pathway respiration. **D** Schematic diagram of the functional position of the AOX respiratory pathway in the respiratory chain. Data are the mean ± SD of five independent experiments. Different lowercase letters above the bars represent significant differences according to post hoc analysis (Tukey’s HSD, *P* < 0.05)

Respiration provides energy that is required for various biological processes in living organisms. Considering that cyanide and H_2_S are both toxic chemicals for cellular respiration and that *CYS*-*C1*-OE exhibited the most prominent salt tolerance under 100 mM NaCl treatment, respiratory parameters, including total respiration (*V*_t_), alternative oxidase pathway respiration (*V*_alt_), and the *V*_alt_/*V*_t_ ratio, were further compared between *CYS*-*C1*-OE and WT plants. The results showed that for WT seedlings, salt stress inhibited *V*_t_ and *V*_alt_ but increased the *V*_alt_/*V*_t_ ratio, indicating that the compensatory effect of AOX is more prominent when cytochrome pathway respiration is inhibited by salt stress (Fig. 4). In comparison, the *CYS*-*C1*-OE seedlings showed higher *V*_t_ and *V*_alt_ than the WT seedlings under the same NaCl concentration conditions, although their respiration rates were also inhibited by salt stress (Fig. 4A, B). Second, increased *V*_alt_/*V*_t_ ratios were also observed in salt stress-treated *CYS*-*C1*-OE seedlings (Fig. 4C). It is interesting to note that the *V*_alt_/*V*_t_ ratios were higher in WT seedlings than in *CYS*-*C1*-OE seedlings; however, regarding *V*_t_ and *V*_alt_, *CYS*-*C1*-OE seedlings, but not WT seedlings, exhibited the strongest respiration rates under salt stress conditions (Fig. 4A, B). Further study showed that the expression of the *AtAOX1a* gene was significantly induced by salt stress, followed by that of *AtAOX1b*, *AtAOX2*, and *AtAOX1c*. In addition, the levels of *AOX* gene expression in the *CYS*-*C1*-OE seedlings were higher than those in the WT seedlings, especially after the 7^th^ day of salt stress (Fig. S5). These results indicate that the AOX pathway plays an important role in CAS overexpression-mediated plant resistance to stress conditions.

**Fig. 5:**
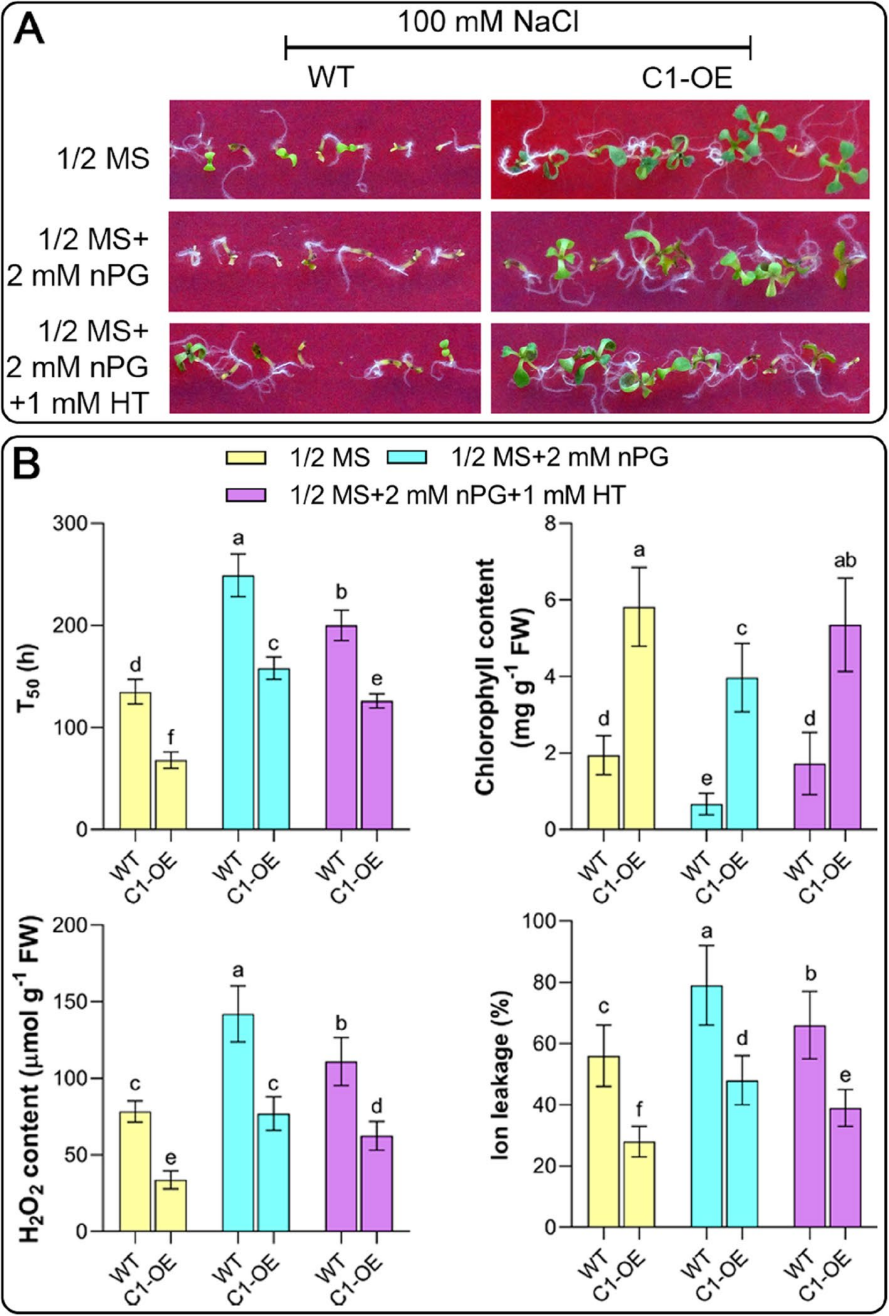
Effects of AOX inhibitor and H_2_S scavenger on salt stress tolerance in Arabidopsis seedlings. For this experiment, seeds were sown on 1/2 MS medium with 100 mM NaCl with or without 2 mM nPG (AOX inhibitor) or 1 mM HT (H_2_S scavenger). **A** The salt stress adaptation of WT and C1-OE seeds after ten days of treatment is shown. **B** The germination speed (T_50_), chlorophyll content, H_2_O_2_ content, and ion leakage were compared between WT and C1-OE seeds. Data are the mean ± SD of five independent experiments. Different lowercase letters above the bars represent significant differences according to post hoc analysis (Tukey’s HSD, *P* < 0.05)

To further confirm the above hypothesis, we next investigated the effects of AOX inhibition on salt stress tolerance in both WT and *CYS*-*C1*-OE seedlings. As shown in Fig. 5, the application of AOX inhibitors (2 mM nPG) reduced the salt stress resistance of WT and *CYS*-*C1*-OE seedlings, although the latter still showed better stress adaptation. The germination speed (T_50_) was prolonged by AOX inhibition, concomitant with decreased chlorophyll contents and increased oxidative damage (e.g., higher H_2_O_2_ contents and ion leakage) (Fig. 5A). In addition, we added a H_2_S scavenger (1 mM HT) to the medium containing the AOX inhibitor and found that the growth of the treated seedlings improved but remained worse than that of the seedlings without the AOX inhibitor (Fig. 5A); however, under such conditions, the germination speed was restored and the oxidative damage was reduced to some extent. These results suggest that AOX plays a vital role in *CAS*-OE-mediated salt stress tolerance and that, in addition to cyanide, H_2_S is also an important factor affecting respiratory homeostasis and the interaction between CAS and AOX.

## Discussion

**Fig. 6:**
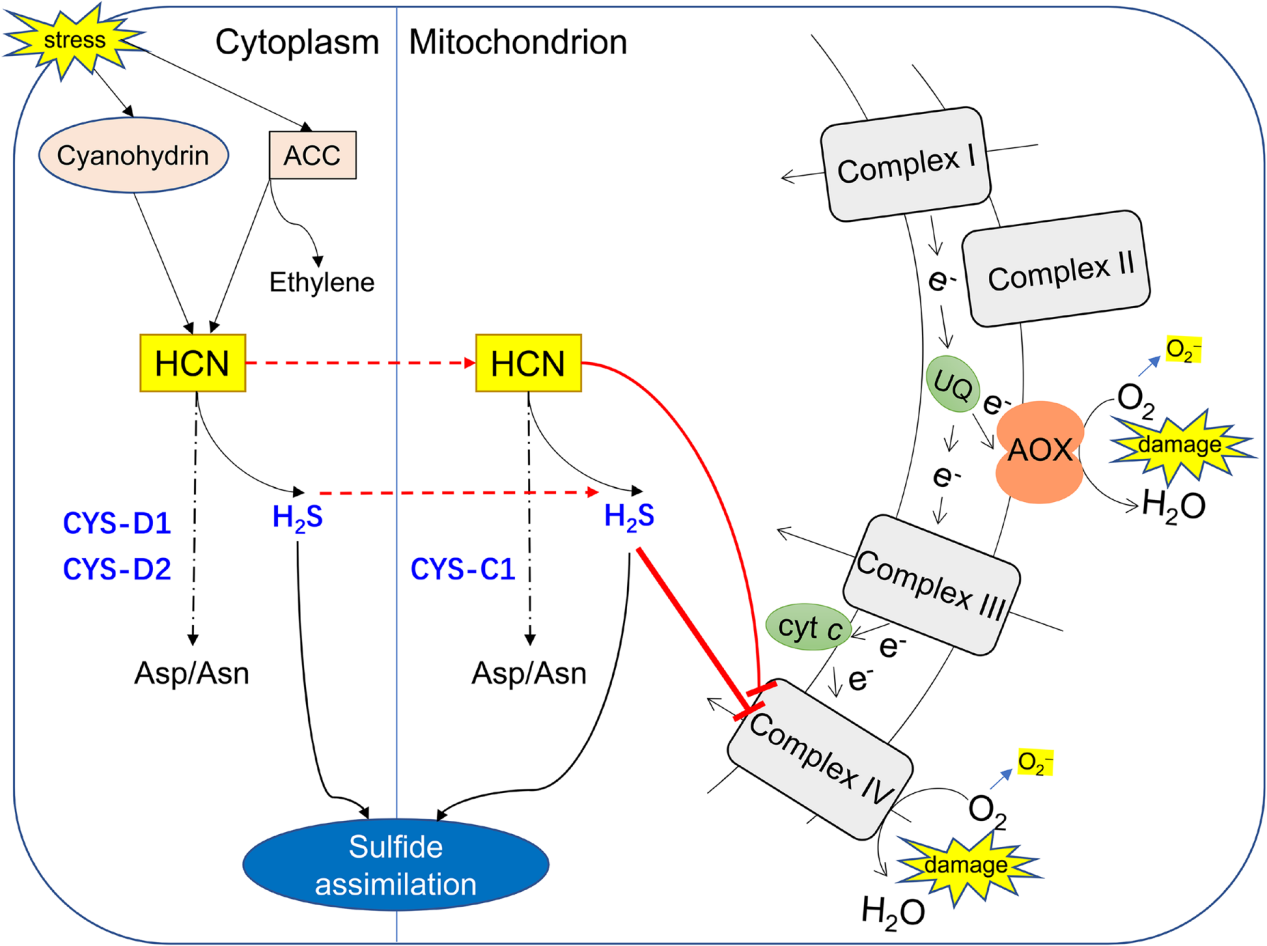
Roles of the *CAS* gene family and AOX in the plant response to stress conditions. In Arabidopsis, environmental stress, such as salt stress, leads to cyanide accumulation in the cytoplasm due to the hydrolysis of cyanohydrin and as a byproduct of ethylene synthesis. Previous studies have shown that CYS-C1 plays a major role in cyanide detoxification in the mitochondria, while CYS-D1 and CYS-D2 play a role in the cytoplasm [19]. The results of this study indicate that CYS-C1 is a key member of the *CAS* gene family involved in cyanide detoxification. Notably, AOX plays an active role in respiratory homeostasis when plants are exposed to stress conditions, especially when the cytochrome *c* pathway is damaged by adverse conditions and toxic chemicals such as HCN and H_2_S. Therefore, it can be concluded that CAS and AOX synergistically contribute to the tolerance of Arabidopsis seeds and seedlings to salt stress

In the present study, we found that overexpression of *CAS* genes contributes to enhancing seed and seedling resistance to salt stress conditions. The results indicated that *CAS* family genes were involved in cyanide detoxification and that *CYS-C1* played a prominent role in this process under normal conditions and different NaCl concentrations. Interestingly, under normal conditions, the essential activity of CAS is able to counteract cyanide production in cells during seed germination, which is consistent with our previous findings in tobacco [11]. However, when seeds were subjected to salt stress, higher levels of cyanide accumulated and higher CAS activities were required to resist stress conditions. Considering that CYS-C1 is located in mitochondria while CYS-D1 and CYS-D2 are located in the cytoplasm, it is plausible that CYS-C1 plays the most important role in salt tolerance by maintaining mitochondrial homeostasis (Fig. 6).

It is worth noticing that overexpression of *CAS* genes helps to enhance the adaptation of seeds to salt stress, which is related to the reduction of cyanide accumulation and oxidative damage, but these benefits seem to require the help of AOX. AOX has long been known as a "stress protein" that responds to intrinsic and extrinsic stresses and contributes to mitochondrial homeostasis [13]. In fact, AOX not only responds to oxidative damage but is also responsible for the incomplete detoxification of cyanide by CAS. Notably, even in the case of *CAS* overexpression, cyanide detoxification was not timely and effective as can be concluded from the detection of cyanide residues in *CAS*-OE seedlings. As a small molecular gaseous compound, the rapid movement of cyanide within and between cells may also lead to untimely cyanide metabolism. Therefore, this might be one of the reasons why AOX needs to assist CAS in mitigating cyanide toxicity. In addition to the beneficial effect of AOX on CAS cyanide detoxification, overexpression of CAS also contributes to alleviating respiratory depression and helping maintain AOX protein function. In this study, the data showed that the ratio of AOX pathway respiration to total respiration increased significantly after plant exposure to salt stress (Fig. 4). Nevertheless, consistent with previous studies [21], it is clear that induction of AOX is insufficient to reduce damage caused by stress and toxic chemicals, as we observed inhibition in the growth of both WT and *CAS*-OE seedlings. Importantly, in contrast, *CAS*-OE seedlings had stronger salt resistance than WT seedlings, and their AOX pathway respiration was significantly higher than that of WT, which further demonstrates that CAS is beneficial for AOX action. Altogether, the interaction of CAS and AOX to enhance plant viability is evident in Arabidopsis.

The novelty of this study is that H_2_S should be considered as another key mediator linking CAS and AOX, in addition to cyanide. Based on the metabolic process, H_2_S is produced in CAS-catalyzed cyanide detoxification [22]. H_2_S is also a toxic respiratory depressant before it is rapidly converted into other chemicals (e.g., cysteine). This may explain why AOX is important for cellular respiration under normal and salt stress conditions, even though CAS has been shown to detoxify cyanide. In our study, however, H_2_S accumulated in WT and *CAS* overexpressing materials with increasing salt concentration, but the amount of H_2_S in *CAS*-OE samples was generally lower than that in WT samples. Although it is not well understood why the decrease in cyanide did not cause an increase in H_2_S according to the metabolic relationship between cyanide and H_2_S mentioned above. But it is worth noticing that the substrate for cyanide metabolism is cysteine, and one of the synthetic substrates for cysteine is H_2_S. Therefore, cysteine and H_2_S form a cyclic pathway during cyanide metabolism [23]. It has been emphasized that the homeostasis of cysteine profoundly affects plant growth and defense responses to abiotic stresses [24, 25]. Some reports have stated that cyanide detoxification and sulfide assimilation are closely linked and mutually reinforcing [21, 23]. Then, previous work has allowed us to propose that the rapid metabolism of cyanide in *CAS*-OE plants also indirectly accelerates the assimilation of H_2_S to maintain cysteine homeostasis, as well as to replenish the substrate cysteine required for cyanide metabolism. It has also been speculated that CAS enzymes, in addition to cyanide detoxification, may also be involved in the process of converting H_2_S to cysteine [18, 23]. Given this, more studies are needed to confirm this hypothesis in the future, such as enzyme kinetic analysis.

In addition to acting on cyanide and H_2_S detoxification, it is evident that CAS and AOX contribute to the antioxidant enzyme system during plant resistance to salt stress. In the present study, salt stress increased the rise in intracellular ROS and MDA in WT, *CAS*-OE and *cas* mutants (Fig. 1), indicating that oxidative damage was caused to cells in the early stage of stress, and then the intracellular antioxidant system was further stimulated. This finding is consistent with the results of many previous studies showing that stress rapidly activates the antioxidant enzyme system [1, 26, 27]. However, *CAS*-OE seedlings exhibited higher antioxidant enzyme activity than WT seedlings (Fig. S4), which may be one of the reasons why *CAS*-OE seedlings showed stronger salt tolerance. Importantly, antioxidant enzyme activities also decreased in *CAS*-OE seedlings as the salt concentration increased to 200 mM NaCl, which may be attributed to more severe cell damage caused by high-salt conditions; after all, cyanide and H_2_S accumulation also increased under such conditions. In other words, to mitigate oxidative damage in cells, CAS and AOX must respond (be stimulated) quickly and act synergistically in cyanide detoxification and cellular respiration stabilization, thereby contributing to the plant's anti-stress response. In comparison, overexpression of *CAS* was more conducive to the detoxification of plant cyanide and other toxic substances and prevented further cellular damage. In addition, it is certain that highly active AOX and antioxidant enzymes are necessary for CAS to enhance the salt tolerance of plants.

## Conclusions

In summary, overexpression of *CAS* genes play a positive role in salt stress tolerance in *Arabidopsis thaliana*. Among these genes, the *CYS-C1* gene is a key member of the *CAS* gene family that is involved in most cyanide detoxification processes and helps mitigate the toxicity of cyanide to cellular respiration. Additionally, AOX plays an important synergistic role in CAS-mediated salt resistance, and a lack of AOX impairs salt tolerance in Arabidopsis, including in *CAS*-OE plants. In addition to cyanide, H_2_S accumulation and oxidative damage caused by salt stress also trigger the necessity for the auxiliary role of AOX. Further studies are needed to better understand sulfide assimilation and its regulation when plants are exposed to unfavorable conditions.

## Methods

### Plant material and growth conditions

Seeds of Arabidopsis including wild-type (WT; Columbia-0, Col-0) and *CAS* gene overexpressing and T-DNA insertion mutants were surface-sterilized in 20% (v/v) commercial bleach for 20 min, followed by six washes with sterile distilled water. The seeds were stratified for 48 h at 4 °C before sowing onto 1/2 MS agar plates with 16 h of light (approx. 120 μmol m^–2^ s^–1^) at 22 °C and 8 h of the dark at 18 °C.

Plants were sourced as follows: The Arabidopsis Col-0 and *CAS* gene family T-DNA insertion mutant seeds including *cys-c1* (NASC ID: N681233), *cys-d1* (NASC ID: N592696), and *cys-d2* (NASC ID: N663434) were obtained from Nottingham Arabidopsis Stock Centre (NASC) and verified by Prof Fei XU and Prof Lu-Lu Yu, who work at the Applied Biotechnology Center, Wuhan University of Bioengineering. In this study, T-DNA verification primers were from SALK (http://signal.salk.edu/tdnaprimers.2.html) to identify the correct seeds for subsequent experimental studies (data not shown for validating mutant seeds).

To generate the *CAS* overexpressing plants, cDNA fragments of At3g61440 (CYS-C1), At3g04940 (CYS-D1), At5g28020 (CYS-D2) including ORF sequence were amplified by high fidelity DNA polymerase and cloned into pBI121 vectors carrying 35S promoter. The primers used for *CAS* gene amplification are shown in Table S1. Plants overexpressing *CAS* genes were transformed by the floral-dip method and transgenic lines were selected on media containing 80 µM kanamycin (Sigma, St Louis, MO, USA). The T_2_ generation with higher gene overexpression (CYS-C1-OE-2, CYS-D1-OE-8, and CYS-D2-OE-6) was used in this study (Fig. S1).

### Salt and chemical treatments

For salt stress treatment, seeds from the WT, *CAS* genes overexpression and T-DNA insertion mutants were plated onto 1/2 MS agar plates, supplied with different concentrations of salt solutions (50, 100, and 200 mM NaCl). Of these, plants were considered to suffer from lower (50 mM NaCl), medium (100 mM NaCl) and higher (200 mM NaCl) concentrations of salt stress. Then, the germination ability and germination rate (T_50_) were recorded and compared between different samples under normal and salt stress conditions.

To further compare the differences in salt resistance after germination, 3-week-old seedlings were used for salt stress treatment. Salt solutions of different concentrations (50, 100, and 200 mM NaCl) were poured into the soil, and then the same concentrations of salt solutions were added to the tray. Repeat the salt treatments every two days and change the salt solution in the tray at the same time.

To inhibit the activity of the AOX respiratory pathway, 2 mM n-propyl gallate (nPG; AOX inhibitor) was added to the 1/2 MS medium with or without salt stress. For the effect of H_2_S scavenging on seed resistant to salt stress, 1 mM hypotaurine (HT) was used [28].

The seeds or seedlings were placed under conditions of 16 h of light (approx. 120 μmol m^–2^ s^–1^) at 22 °C and 8 h of the dark at 18 °C, 70% relative humidity.

### Cyanide quantification

A total of 0.1 g of plant tissue was homogenized using a mortar and pestle with liquid nitrogen and resuspended in cold borate-phosphate extraction buffer (2 mL g^−1^ fresh weight) containing 27 mM sodium borate and 47 mM potassium phosphate, pH 8.0. Homogenates were centrifuged at 15, 000 g for 15 min at 4 °C. Extracted cyanide was subsequently quantified by reverse-phase HPLC after derivatization with 2, 3-naphthalenedialdehyde to form a 1-cyano-2-alkyl-benz[f]isoindole derivative by previously described methods [16]. The HPLC system included a Binary HPLC pump (Waters 1525), autosampler (Waters 2707), and fluorescence detector (Waters 2475). The cyano-alkyl-benz[f]isoindole derivative was separated on an RP 18 (150 mm × 3.9 mm, 5 mm; Waters) column. The mobile phase consisted of a mixture of acetonitrile and 0.1% trifluoroacetic acid in water (28:72 v/v) and was delivered isocratically at a flow rate of 1 mL/min. The injection volume was 10 µL [12].

### Hydrogen sulfide quantification

Hydrogen sulfide (H_2_S) quantification was carried out according to the methods of Baudouin et al. [28]. Samples (~ 50 mg) were ground in liquid nitrogen and powders were resuspended in 500 µL of 100 mM potassium phosphate buffer (pH 7.0) containing 10 mM EDTA. Following centrifugation (14,000 g, 4 °C, 15 min), H_2_S content from 100 µL supernatant was measured in a final volume of 2 mL containing 100 mM potassium phosphate buffer (pH 7.0), 10 mM EDTA, 0.2 mM 5,5′-dithiobis (2-nitrobenzoic acid). The assay mixture was incubated at room temperature for 5 min and the absorbance was determined at 412 nm using a spectrophotometer (TU1800 spectrophotometer, P-general Limited Company, Beijing, China). H_2_S quantity was deduced from a standard curve obtained with known NaHS concentrations (0 ~ 10 µM).

### Respiration measurements

Respiration of Arabidopsis seedlings was carried out by previously described methods [11] with some modification. Approximately 50 mg of samples were collected and transferred into air-tight cuvettes containing 2 mL of phosphate-buffered saline (pH 7.5), and oxygen uptake was measured as a decrease of oxygen concentration in the dark using a Clark-type electrode (Chorolab-2; Hansatech, King’s Lynn, UK). Inhibitors of the cytochrome *c* pathway (1 mM KCN) and the AOX pathway (2 mM n-propyl gallate; nPG) were used. Total respiration (*V*_t_) is defined as oxygen uptake rate by samples without any inhibitor. AOX pathway mediated cyanide-resistant oxygen uptake (*V*_alt_) was measured in the presence of 1 mM KCN. The oxygen uptake of cytochrome *c* pathway (*V*_cyt_) was measured in the presence of 2 mM nPG. Residual respiration (*V*_res_) is defined as the oxygen uptake in the presence of both 1 mM KCN and 2 mM nPG. Oxygen uptake by both the AOX pathway (*V*_alt_) and the cytochrome *c* pathway (*V*_cyt_) needs to subtract the residual respiration (*V*_res_), although residual respiration in our experiments was always very low and negligible relative to other respirations.

### Measurement of total chlorophyll contents

Plant tissue total chlorophyll was extracted and measured by previously described methods [29]. Approximately 0.5 g of samples were ground with 5 mL 80% acetone. Following centrifugation (10, 000 g, 4 °C, 15 min), 1 mL supernatant was used to read the absorbance values of chlorophyll *a* and chlorophyll *b* at the wavelength of 663 and 645 nm, respectively, using a spectrophotometer (TU1800 spectrophotometer, P-general Limited Company, Beijing, China). Total chlorophyll contents were calculated and expressed as mg per gram of fresh weight.

### Oxidative damage estimation

The H_2_O_2_ content of seedlings was measured by previously described methods [30]. Approx. 0.5 g of samples were cut into small pieces and homogenized in an ice bath with 5 mL 0.1% (w/v) trichloroacetic acid (TCA). The homogenate was centrifuged at 12, 000 g for 20 min at 4 °C. 0.5 mL of the supernatant was added to 0.5 mL 10 mM potassium phosphate buffer (pH 7.0) and 1 mL 1 M KI. The absorbance of the supernatant was read at 390 nm. The content of H_2_O_2_ was determined by a standard curve.

Electrolyte leakage (EL) was calculated by measuring the conductivity [31]. Approx. 0.5 g of samples were cut into small pieces and transferred to 10 mL deionized water. The conductivity (C1) was measured after standing for 2 h at room temperature. After measuring the conductivity, the samples were boiled for 15 min to achieve 100% ion leakage (C2). Relative conductivity = C1/C2 × 100%.

Lipid peroxidation was estimated by measuring the malondialdehyde (MDA), according to the protocol of Micro MDA Assay Kit (Solarbio, Beijing, China).

### Antioxidant enzymes assay

A crude enzyme was extracted by previously described methods [8]. Approx. 0.5 g of samples were ground with 3 mL ice-cold 25 mM Hepes buffer (pH 7.8) containing 0.2 mM EDTA, 2 mM ascorbate and 2% PVP. The homogenates were centrifuged at 4 °C for 20 min at 12, 000 g and the resulting supernatants were used for the determination of enzymatic activity. Levels of the antioxidant enzyme activities including superoxide dismutase (SOD; E.C. 1.15.1.1), catalase (CAT; E.C. 1.11.1.6), ascorbate peroxidase (APX; EC 1.11.1.11), and guaiacol peroxidase (GPX; EC 1.11.1.9) were assayed according to the methods of Yu et al. [8].

### Total RNA extraction and quantitative real time PCR

Total RNA extraction and quantitative real time PCR (qRT-PCR) were carried out by previously described methods [32]. First-strand cDNA was reverse transcribed from DNase I-treated RNA with oligo (dT) as the primer. qRT-PCR experiments were performed with the ChamQ SYBR qPCR Master Mix (Vazyme Biotech Co., Ltd) in a ABI7500 cycler (Applied Biosystems) with three technical repeats for each sample. Reactions were initiated at 94 °C for 15 min followed by 40 cycles at 94 °C for 30 s, 56 °C for 30 s, and 72 °C for 30 s. The relative quantitation of the target gene expression level was performed using the comparative Ct (threshold cycle) method. The amplification of the *PP2AA3* gene (encoding protein phosphatase 2A subunit A3, At1g13320) was used for an internal control [33]. Primers used for qRT-PCR are listed in Table S2.

## Supplementary Information

**Additional file 1: Table S1.** Primers used for CDS amplification and mutation identification of *CAS *genes. **TableS2.** Primers used for qRT-PCR. **Fig. S1.** Relative expression of *CAS*-OE lines. **Fig. S2.** Effects of *CAS *gene mutants on seed germination and growth. **Fig. S3.** *CAS *gene mutations impair salt stress resistance in Arabidopsis seedlings. **Fig. S4.** Comparison of antioxidant enzyme activity between WT and *CAS *overexpressing seedlings. **Fig. S5.** Changes in the expression of *AOX *family genes.

## Data Availability

The data that support the findings of this study are available from the corresponding author upon reasonable request.

## Abbreviations

AOX: Alternative oxidase
APX: Ascorbate peroxidase
CAS: Cyanoalanine synthase
CAT: Catalase
EL: Electrolyte leakage
GPX: Guaiacol peroxidase
H_2_S: Hydrogen sulfide
HT: Hypotaurine
MDA: Malondialdehyde
nPG: n-Propyl gallate
qRT-PCR: Quantitative real time PCR
SOD: Superoxide dismutase
