# Supplementary material for: The role of cyanoalanine synthase and alternative oxidase in promoting salt stress tolerance in Arabidopsis thaliana
Source: BMC Plant Biol. 2023 Mar 27;23:163. doi: 10.1186/s12870-023-04167-1 (PMC10041793; doi:10.1186/s12870-023-04167-1)
Supplement: Supplementary file 1 — Additional file 1: Table S1. Primers used for CDS amplification and mutation identification of CAS genes. TableS2. Primers used for qRT-PCR. Fig. S1. Relative expression of CAS-OE lines. Fig. S2. Effects of CAS gene mutants on seed germination and growth. Fig. S3. CAS gene mutations impair salt stress resistance in Arabidopsis seedlings. Fig. S4. Comparison of antioxidant enzyme activity between WT and CAS overexpressing seedlings. Fig. S5. Changes in the expression of AOX family genes. [file 12870_2023_4167_MOESM1_ESM.pdf]

Supporting Information

Table S1 Primers used for CDS amplification and mutation identification of *CAS* genes

Gene name	Gene ID or NASC ID	Forward Primer (5'→3')	Reverse Primer (5'→3')
CYS-C1	At3g61440	CG**GGATCC**CGTCTTCACTCTCTTG TCTCTTC	C**GAGCTC**GGTCAGTCTTCCTTC ATCAATC
CYS-D1	At3g04940	CG**GGATCC**CGCGACAGTCTTCTTC TTCTTCTT	C**GAGCTC**GGAGGCTAGAGATTA GAGATCATAC
CYS-D2	At5g28020	CG**GGATCC**CGATGGAAGATCGGTG CTTGA	C**GAGCTC**GTCTTGGACTCTTGG AACGAA
cys-c1	N681233	GGTTTCTGGCCTTGTCCTTAG	GGCGTTTTCCCAATAAGCTAC
cys-d1	N592696	TTCACCAAAACGAACCAAGTC	AACCCGCAAGTATGGACTCTC
cys-d2	N663434	TTCAATAGCCTCTTCACCTGC	TGAGATGATGGAGCCTTGTTC

Note: The bold letters are restriction enzyme cleavage sites. *Bam*HI: CG**GGATCC**CG; *Sac*I: C**GAGCTC**G.

Table S2 Primers used for qRT-PCR

Gene name	Accession number	Forward Primer (5'→3')	Reverse Primer (5'→3')
CYS-C1	AY093094	CCAATCACTCCTCCTCTAA	AACATACGCTTCACAACCT
CYS-D1	AY136420	TCCTCACAATGCCCTCA	TGCCTCCACTTATTACCG
CYS-D2	AY124831	GATTCATTACCGAACCACG	GCCTCTTCACCTGCCACT
AtAOX1a	NM_113135	GAGCAAAGTGGAGGATGG	CACGACCTTGGTAGTGAATAT
AtAOX1b	NM_113134	AAAACTACGGAGGAGAAAGG	TGAACGGCAATCACAAGA
AtAOX1c	NM_113678	GAAGTAGCGAAACCCAAAT	TCAGCACGAACCACCAT
AtAOX2	NM_125817	ATTTGACGGTAAAGAAGGG	TGGGAATACGGAGGAGC
PP2AA3	NM_101203	GGCTTTCTATCATTGCTCGTG	GCATACTCAACCCCTCCTACAT

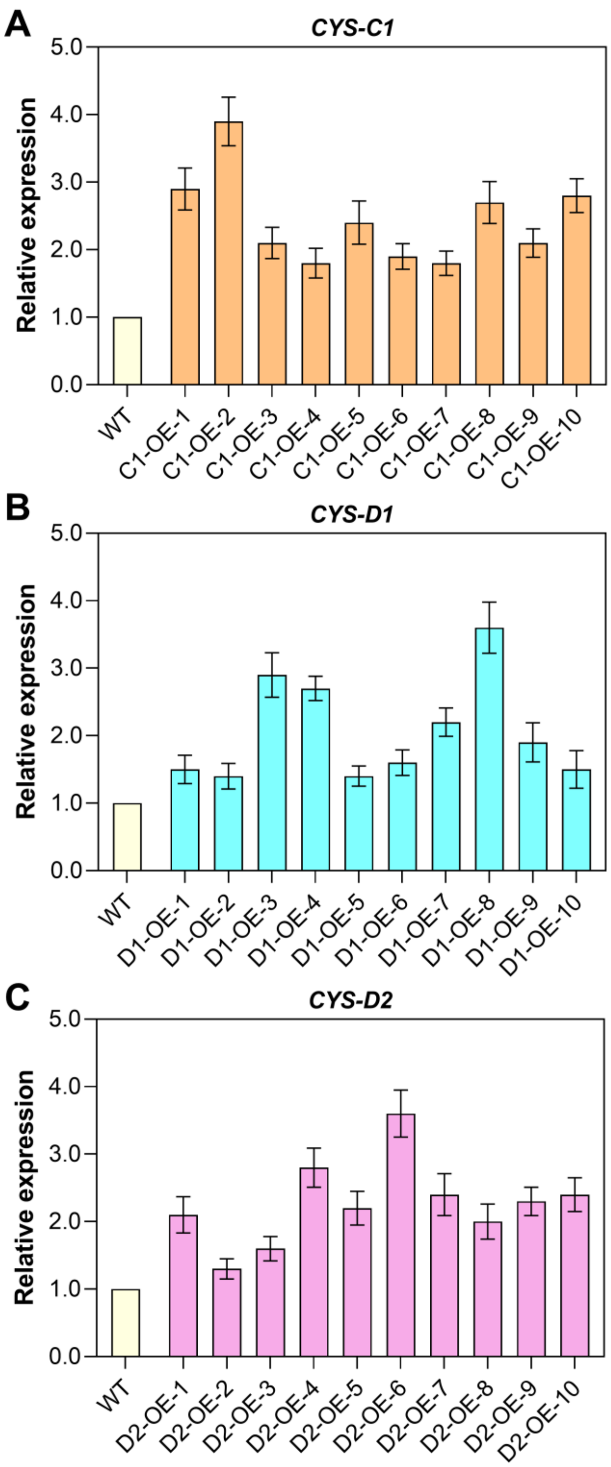

Fig. S1 Relative expression of *CAS*-OE lines. For this experiment, 3-week-old seedlings were used for gene expression analysis. The mRNA of Ten T_2 generations of transgenic plants were extracted and the cDNAs were used for qRT-PCR. In this study, transgenic plants C1-OE-2, D1-OE-8, and D2-OE-6 were selected for salt stress resistance analysis.

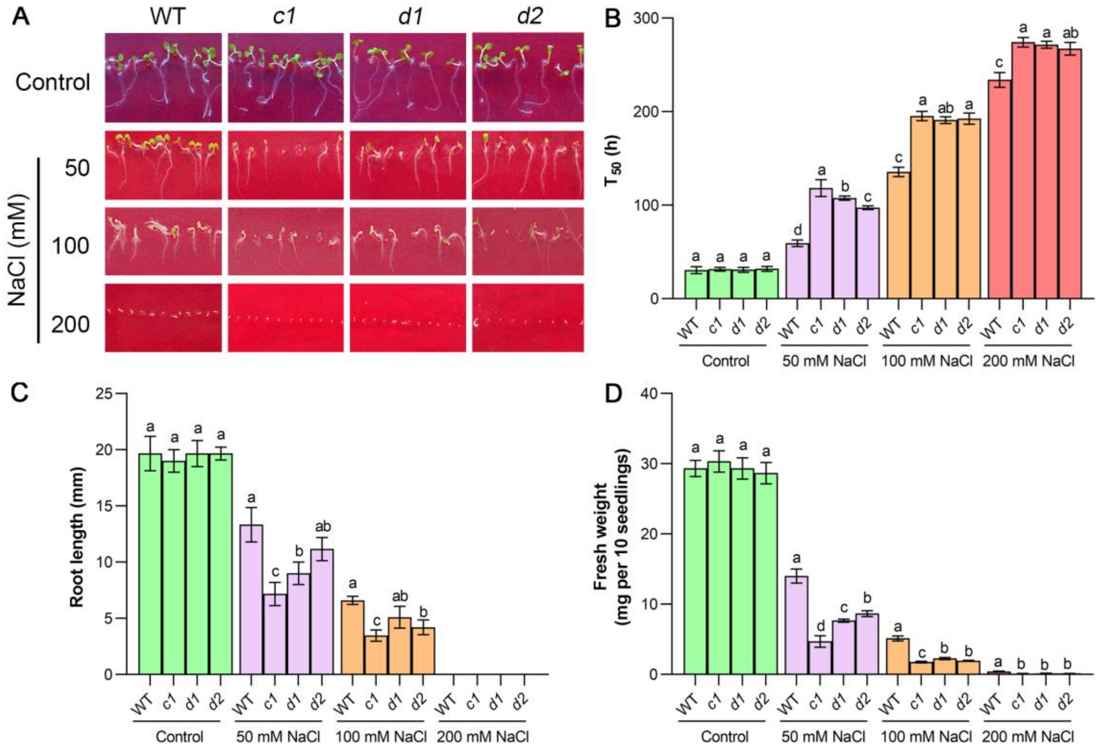

Fig. S2 Effects of *CAS* gene mutants on seed germination and growth. WT and mutant seeds were sown on 1/2 MS medium with different concentrations of NaCl. After seeds are incubated for ten days, the phenotypes after germination are shown (A), the germination speed (T_{50}) (B), root length (C), and fresh weight (D) were compared between different samples under normal and salt stress conditions. Data are the mean ± SD of five independent experiments. Different lowercase letters above the bars represent significant differences according to *post hoc* analysis (Tukey's HSD, $P<0.05$). The following abbreviations for *CAS* gene mutants were used for labeling in this figure and the following figures: *c1*, *cys-c1*; *d1*, *cys-d1*; *d2*, *cys-d2*.

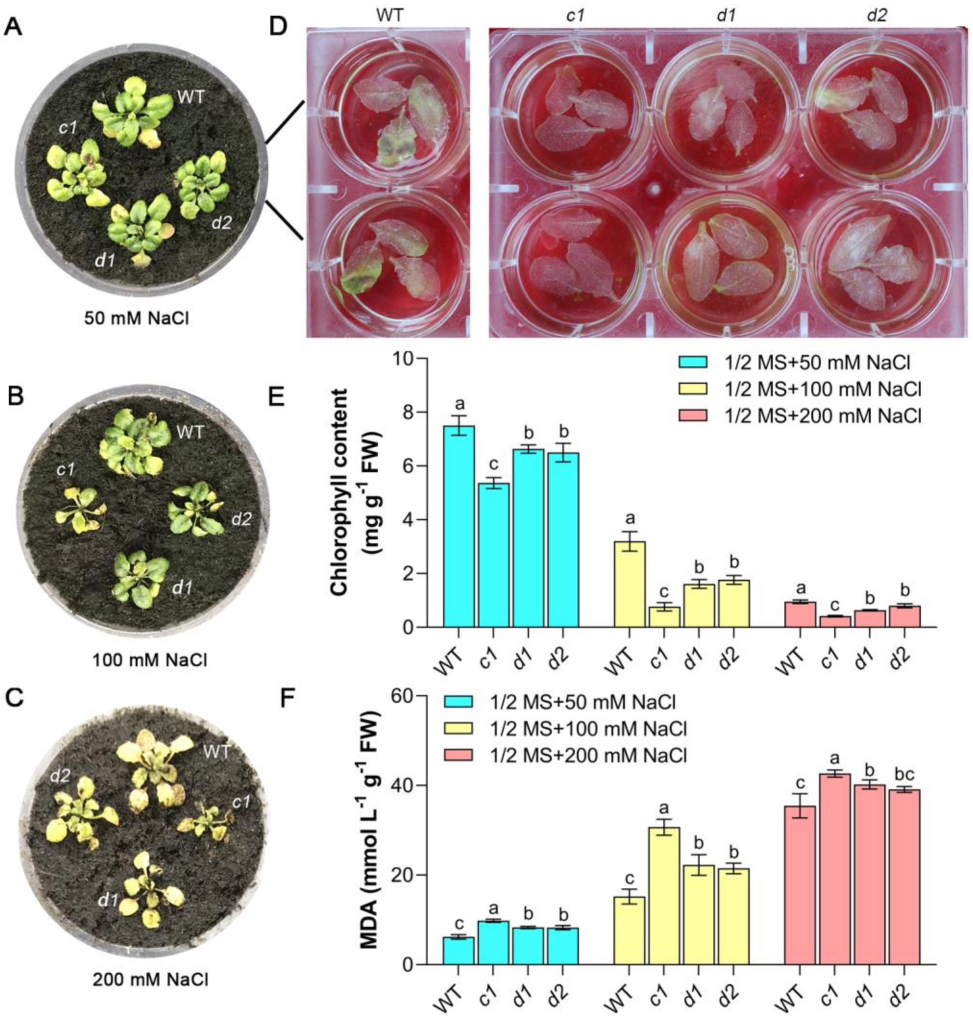

Fig. S3 *CAS* gene mutations impair salt stress resistance in Arabidopsis seedlings. For this experiment, 3-week-old seedlings were irrigated with different concentrations of NaCl. (A-C) After 14 days of salt stress treatments, the seedling phenotypes are shown, and the chlorophyll content (E) and MDA content (F) were measured. (D) To further confirm the differences in salt resistance between WT and *cas* mutants under 50 mM NaCl condition, leaves were collected and placed in the same concentration of the saline solution and the phenotypes are shown after 14 days of stress treatment. Data are the mean ± SD of five independent experiments. Different lowercase letters above the bars represent significant differences according to *post hoc* analysis (Tukey's HSD, $P<0.05$).

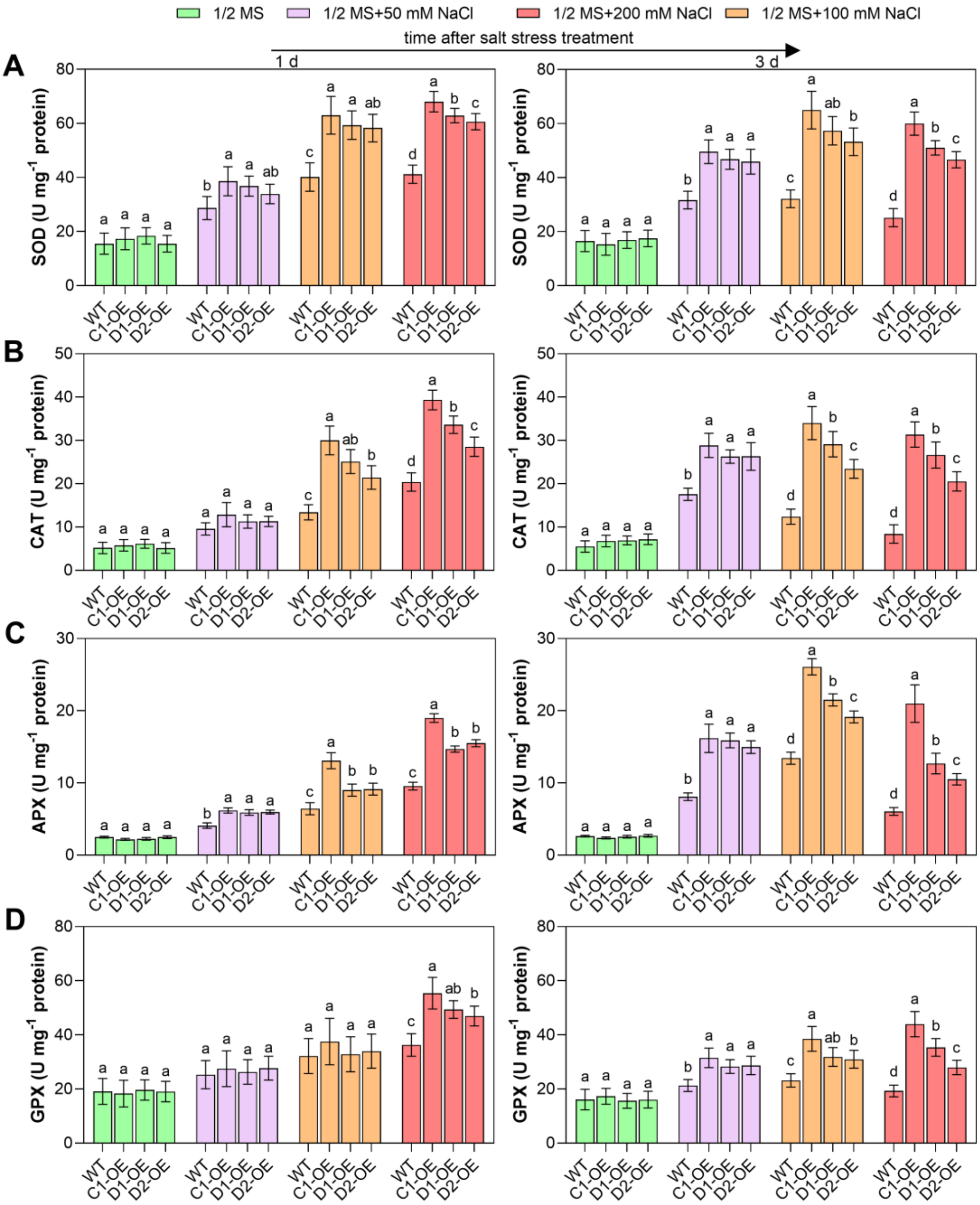

Fig. S4 Comparison of antioxidant enzyme activity between WT and *CAS* overexpressing seedlings. 3-week-old seedlings were treated with different concentrations of NaCl, and the activities of SOD (A), CAT (B), APX (C), and GPX (D) were then measured after salt stress treatment for 1 d and 3 d. Data are the mean ± SD of five independent experiments. Different lowercase letters above the bars represent significant differences according to *post hoc* analysis (Tukey's HSD, *P*<0.05). The following abbreviations for *CAS* gene overexpression were used for labeling in this figure: C1-OE, CYS-C1-OE; D1, CYS-D1-OE; D2, CYS-D2-OE.

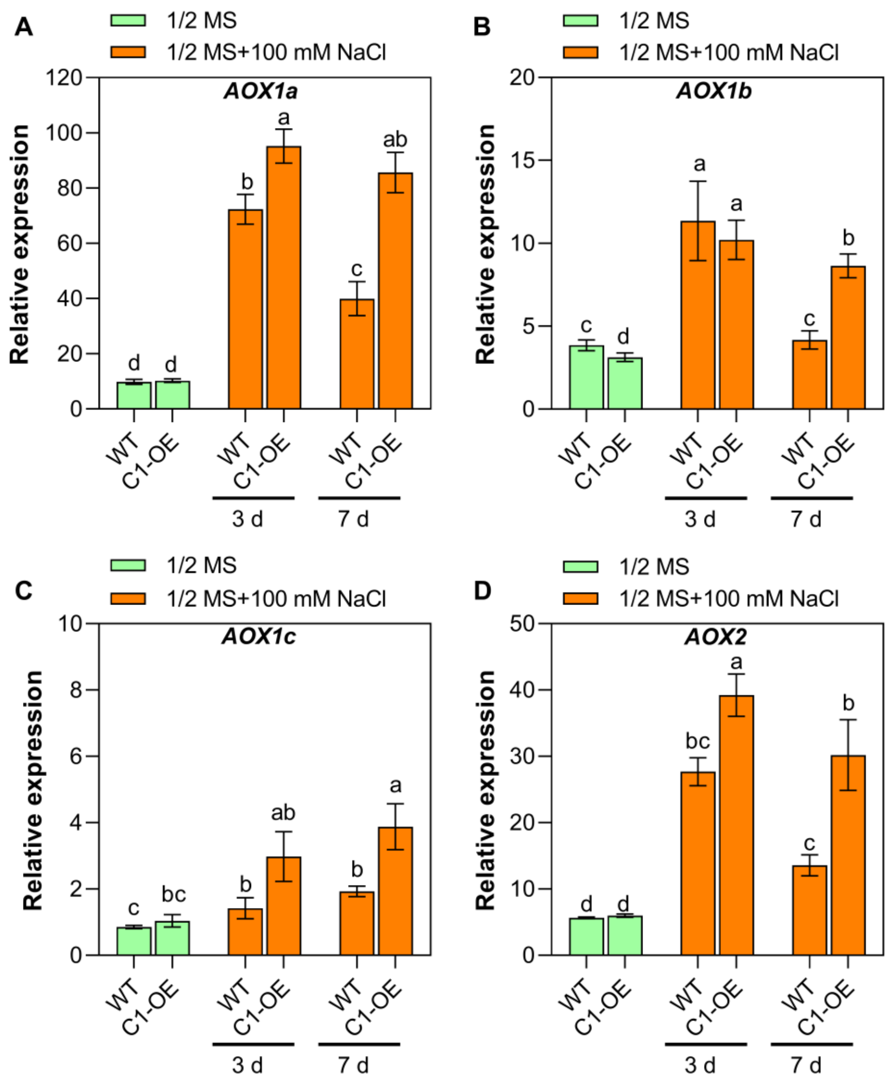

Fig. S5 Changes in the expression of *AOX* family genes. For this experiment, 3-week-old seedlings were treated with or without 100 mM NaCl, and then the relative gene expression of *AOX1a* (A), *AOX1b* (B), *AOX1c* (C), and *AOX2* (D) was compared between WT and *CYS-C1* overexpressing (C1-OE) seedlings by qRT-PCR. Data are the mean ± SD of five independent experiments. Different lowercase letters above the bars represent significant differences according to *post hoc* analysis (Tukey's HSD, $P<0.05$).
